# The Effect of Through-Silicon-Via Thermal Stress on Metal-Oxide-Semiconductor Field-Effect Transistor Properties Under Cooling to Ultra-Low Temperatures

**DOI:** 10.3390/mi16020221

**Published:** 2025-02-15

**Authors:** Wenting Xie, Xiaoting Chen, Liting Zhang, Xiangjun Lu, Bing Ding, An Xie

**Affiliations:** 1 Key Laboratory of Functional Materials and Applications of Fujian Province, School of Material Science and Engineering, Xiamen University of Technology, Xiamen 361024, China; m18773043996@163.com (W.X.); xiaotingchen42@163.com (X.C.); zlttan90@163.com (L.Z.); 2 College of Materials Science and Technology, Nanjing University of Aeronautics and Astronautics, Nanjing 210016, China; bingding@nuaa.edu.cn

**Keywords:** ultra-low temperature, through-silicon-via, thermal stress, carrier mobility, saturation current, threshold voltage

## Abstract

The thermal through-silicon-via (TTSV) has a serious thermal stress problem due to the mismatch of the coefficient of thermal expansion between the Si substrate and filler metal. At present, the thermal stress characteristics and strain mechanism of TTSV are mainly concerned with increases in temperature, and its temperature range is concentrated between 173 and 573 K. By employing finite element analysis and a device simulation method based on temperature-dependent material properties, the impact of TTSV thermal stress on metal-oxide-semiconductor field-effect transistor (MOSFET) properties is investigated under cooling down from room temperature to the ultra-low temperature (20 mK), where the magnitude of thermal stress in TTSV is closely associated with the TTSV diameter and results in significant tension near the Cu-Si interface and consequently increasing the likelihood of delamination and cracking. Considering the piezoresistive effect of the Si substrate, both the TTSV diameter and the distance between TTSV and MOSFET are found to have more pronounced effects on electron mobility along [100] crystal orientation and hole mobility along [110] crystal orientation. Applying a gate voltage of 3 V, the saturation current for the 45 nm-NMOS transistor oriented along channel [100] experiences a variation as high as 34.3%. Moreover, the TTSV with a diameter of 25 μm generates a change in MOSFET threshold voltage up to −56.65 mV at a distance as short as 20 μm. The influences exerted by the diameter and distance are consistent across carrier mobility, saturation current, and threshold voltage parameters.

## 1. Introduction

In the information age, chips are subject to increasingly stringent performance requirements, necessitating a higher density of components [1]. Three-dimensional packaging enables the vertical stacking of multiple chips, enhancing integration with invariant chip area, and facilitates the construction of intricate systems that comprise diverse materials, structures, and devices with varying functionalities [2,3,4]. Three-dimensional packaging is also beneficial for superconducting quantum chips that face multiple challenges. Yost and Rosenberg’s approach utilizing 3D chip stacking not only alleviates wiring congestion, resulting from quantum chip expansion, but also reduces the 2D occupied space of chips while introducing additional dimensions for qubits [5,6,7]. The increased integration degree in 3D packaging also leads to the substantial rise in power density per unit volume, where the heat generated by the multi-layered integrated chips needs to be transferred to the heat sink through adjacent and bonded layers [8,9,10]. However, in spite of the space saved, the ultra-thin chips in the 3D packaging suppress the parallel thermal diffusion effect [11,12].

Through-silicon-via (TSV) is an effective methods for addressing the current thermal challenges in 3D-integrated circuits [13,14,15,16]. Thermal TSV (TTSV) integrated in 3D-integrated circuits accomplishes heat transfer to the heat sink in the perpendicular direction, serving as dedicated thermal paths, rather than transmitting electrical signals, significantly reducing chips temperature. It is noted that the TTSV is also applied in 3D-integrated superconducting quantum chips to dissipate heat from the readout/control circuit [17], keeping the typical operating temperature for the quantum chips at ultra-low temperature of 20 mK. An important problem for the application of TTSV in 3D-integrated circuits is that a mismatch arises between the coefficient of thermal expansion (CTE) of the filling material Cu and that of the substrate Si. This discrepancy can induce thermal stress on the Si substrate, altering the mobility of the substrate material with the piezoresistive effect and impacting the performance of active devices located in proximity to the TTSV [18]. It is reported that the discreteness of ion due to TSV-induced stress exceeds 3% for the 28 nm CMOS devices [19].

Aiming to address the CTE mismatch caused by temperature increasing in traditional 3D packaging, extensive research has been conducted on the generation mechanism and the distribution of TTSV thermal stress in various structures and materials [20,21,22], where the temperature range of studying TTSV thermal stress characteristics and strain mechanism is concentrated between 173 and 573 K. Nevertheless, the typical operating temperature in 3D-integrated superconducting quantum chips with the resonant frequency of 5 GHz is the ultra-low temperature of 20 mK [23]. Thus, there is a gap in the understanding of TTSV behavior under cooling down conditions to ultra-low temperatures, so the goal of this work is to fill this gap. Furthermore, Song et al. validated the impact of material temperature characteristics on TTSV stress through electron backscattering diffraction and finite element analysis (FEA) [24]. Consequently, considering material-specific temperature-dependent properties, this study investigates the influence of TTSV size on thermal stress within the temperature range of 20 mK to 300 K and analyzes the resultant deformation. Then, the FEA is employed to characterize the changes in carrier mobility caused by TTSV-induced stress. Additionally, the ways in which the size of TTSV and the distance between TTSV and metal-oxide-semiconductor field-effect transistor (MOSFET) affect carrier mobility have been examined to optimize the arrangement of neighboring devices. Finally, the effect of TTSV thermal stress on electrical properties of the 45 nm-MOSFET integrated into 3D chips is evaluated.

## 2. Thermal Stress Characteristics of TTSV

### 2.1. Simulation and Analysis Conditions

**Figure 1:**
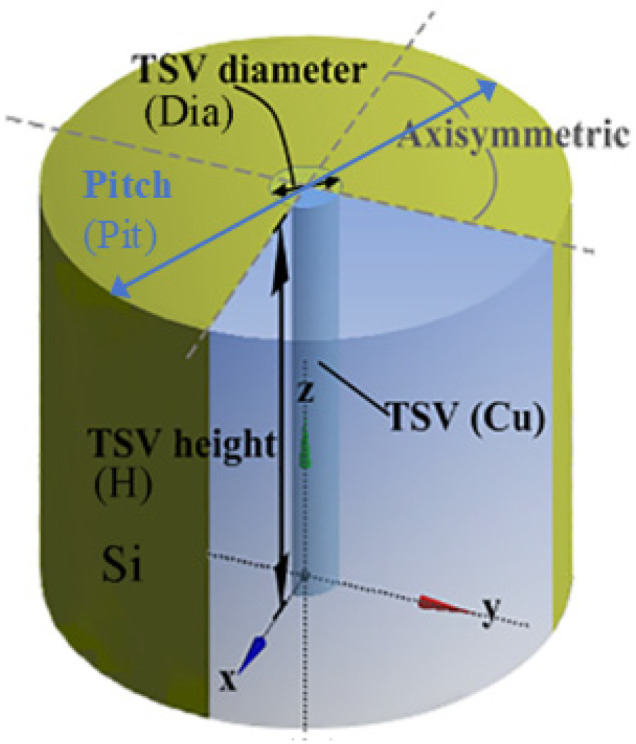
The schematic of TTSV model.

**Figure 2:**
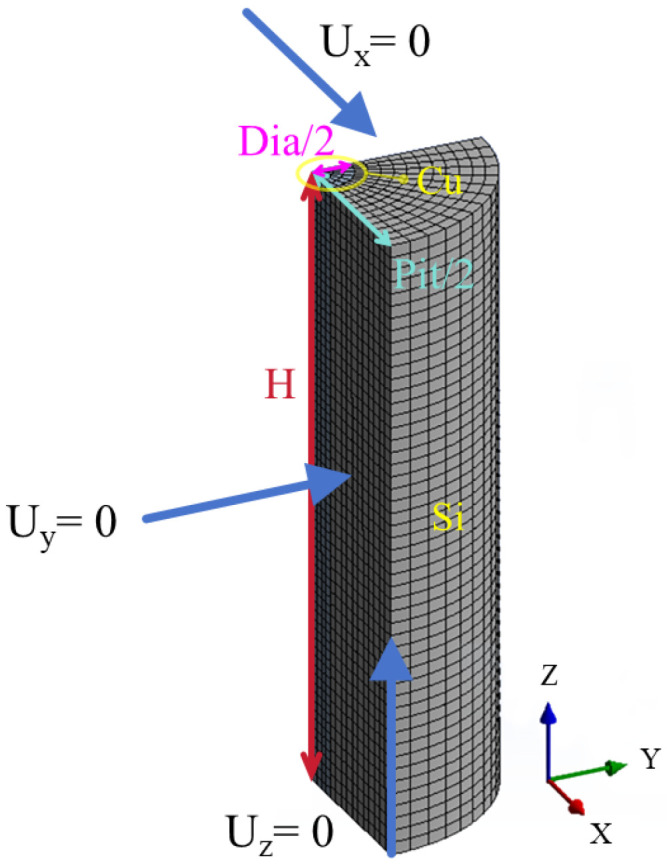
Modeling domain of the unit cell model.

The schematic of the TTSV model is illustrated in Figure 1. The 3D TTSV model is constructed by ANSYS 2022 R1 for transient thermal stress analysis. The thermal stress simulation of TTSV can be transformed into the application of a single TTSV unit in the algorithm analysis based on the linear superposition principle [22]. However, this paper does not provide a detailed analysis of the factors that influence the interaction between TTSVs (such as density and relative angle). Therefore, only a single TTSV unit is analyzed instead of considering the TTSV periodic array. In the 3D model domain, Cu and Si are, respectively, used as the filling material for TTSV and the substrate with the via diameter of Dia, the via height of H, the aspect ratio of Asp (the quotient of H divided by Dia) and the pitch of Pit. Thin insulation and diffusion barrier layers are neglected. By applying symmetry conditions, the model simplifies to 1/4 unit. Using the sweeping method (Figure 2), 103,753 elements are meshed. The normal displacement at two symmetric interfaces is constrained to zero to ensure symmetry, and the *Z*-axis displacement at the bottom interface is limited to zero to reflect the actual constraints imposed on TTSV by the Si substrate [25].

**Figure 3:**
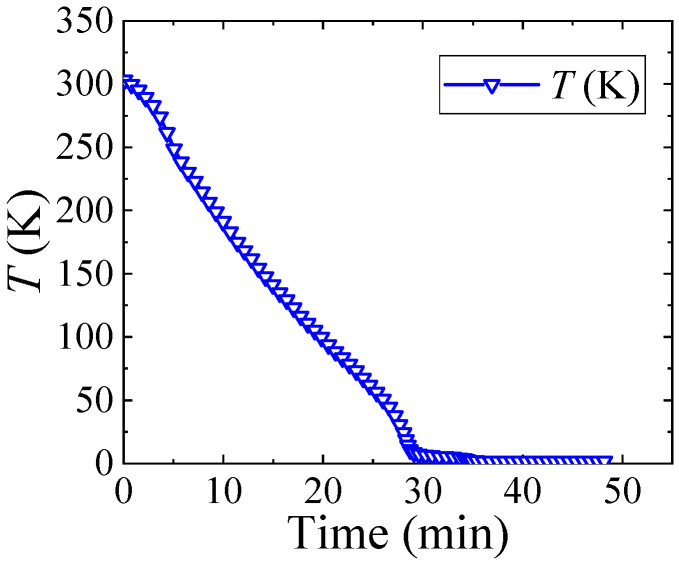
Cooling process of the TTSV model with the PPMS refrigeration system.

**Figure 4:**
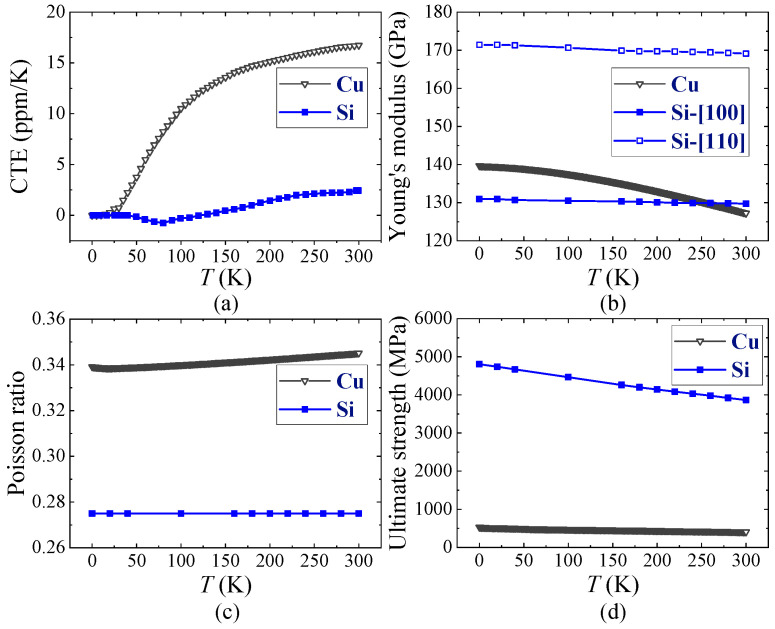
Temperature-dependent material properties of Si and Cu: (**a**) CTE, (**b**) Young’s modulus, (**c**) Poisson’s ratio, and (**d**) ultimate strength.

Cooling starts at 300 K and ends at 20 mK. To comprehensively analyze the process of thermal stress change, transient analysis conditions were configured based on the cooling process of the physical property measurement system (PPMS) (DynaCool system) [26] in Figure 3. When the thermal conductivity of the materials is considered [27,28] and the heat flows across the boundary between dissimilar materials such as Si and Cu, using the thermal boundary resistance of Cu/Si, is 2.5 × 10^−3^ mm^2^ K/W [29], it is found that the temperature distribution within the model remains uniform and synchronizes with the thermal conditions applied to its surface. Because the model contains no power-generating components nor a constant temperature radiator and is small in size, the temperature based on the curve in Figure 3 is assigned to all parts in the TTSV model for temperature control. Since thermal stress depends on material properties that vary with temperature, for a more accurate analysis of the thermal stress behavior, temperature-dependent material properties are considered to increase strain rate sensitivity. The mapping relationship between material characteristics (CTE, Young’s modulus, Poisson’s ratio, and ultimate strength) and temperature is illustrated in Figure 4 [30,31,32,33,34,35,36,37].

### 2.2. Thermal Stress Distribution of TTSV

**Figure 5:**
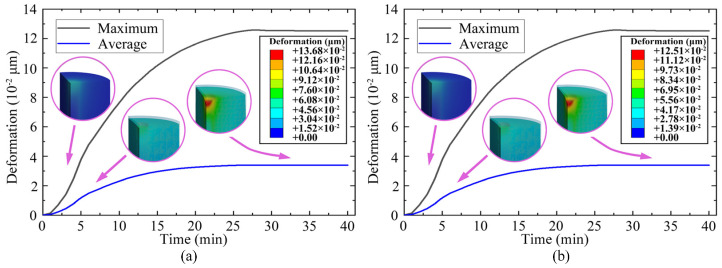
TTSV deformation induced by thermal stress when temperature-dependent material properties are (**a**) not considered and (**b**) considered.

**Figure 6:**
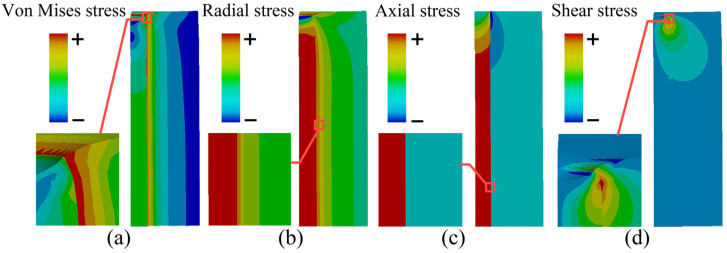
Thermal stress distribution in the TTSV and location of maximum stress: (**a**) von Mises, (**b**) radial, (**c**) axial, and (**d**) shear stress.

The time variation process of TTSV deformation obtained through transient analysis is illustrated in Figure 5. In the deformation comparison of considering and not considering temperature-dependent material properties, the cloud images exhibit consistency in pattern but differ in specific values, where the temperature-dependent material properties make the trend of results remain and enhance the accuracy of the outcomes. Temperature-dependent properties are used for the following analysis. As the temperature decreases, both the mean and maximum deformation gradually grow, particularly with regard to Cu depression. The deformation gradient reaches a peak at the 4~5th min, while the maximum shape variable reaches 12.60 × 10^−^^2^ μm at the 28th min before stabilizing by the 30th min. The stress distribution of the TTSV with a Dia of 25 μm, Asp of 8 and Pit of 110 μm is illustrated in Figure 5. Due to the CTE mismatch between Cu and Si, Cu is restrained by the Si substrate, and the accumulation of the axial contraction of Cu at both ends of the through-via exacerbates the deformation. The maximum von Mises stress for 548.75 MPa is located at Cu-Si interface of TTSV port (Figure 6a) and exceeds the yielding strength, reaching the point where permanent plastic deformation must be considered. As depicted in Figure 6b, the maximum radial stress with 314.97 MPa manifested as the compressive stress appears within the Cu interior near the interface. This is attributed to radial contraction causing squeezing within the Cu interior where stress cannot be fully released, and the closer the Si substrate, the greater the radial constraint. Since Cu shrinks radially after cooling down, it is subjected to radial stretching by the Si substrate at the interface. Also, the axial shrinkage deformation of Cu accumulates at the ends of the via causing the significant radial deformation, intensifying the radial tensile stress at Cu ports. Consequently, the maximum radial tensile stress (161.97 MPa) emerges at the Cu ports close to the interface. As shown in Figure 6c, significant axial deformation emerges at the TTSV ports as a result of cumulative axial contraction deformation in Cu along with a stretching effect on Si, leading to substantial axial tensile stress (195.20 MPa) appearing on the Cu-Si contact surface near both ends of the TTSV. Internal axial shrinkage within Cu prevents the complete release of stress; thus, greater proximity to the Si substrate results in a stronger axial constraint, presenting a maximum compressive axial stress as high as 456.88 MPa near the contact surface in Cu region. The sudden deformation point occurs at the interface of the TTSV ends and exhibits maximum shear stress for 194.30 MPa (Figure 6d), where interfacial delamination is prone to occur. The tension at and near the Cu-Si interface is significant, thereby increasing potential risks.

**Figure 7:**
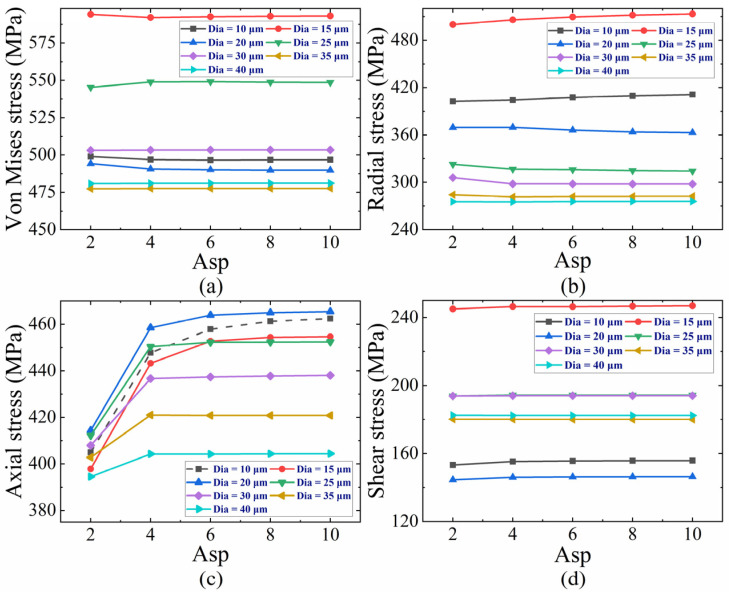
(**a**) Von Mises, (**b**) radial, (**c**) axial, and (**d**) shear stress for various Dias and Asps.

The variations in von Mises, radial, axial, and shear stress with different Dias and Asps are further investigated. Given the difficulty of high Asp in Cu filling, a Dia of 10, 15, 20, 25, 30, 35, and 40 μm is selected along with an Asp of 2, 4, 6, 8, and 10. As depicted in Figure 7, the other stresses except for axial stress show insensitivity for the Asp. The von Mises stress is more dependent on the Dia and reaches the maximum value at 15 μm (Figure 7a), which poses a reliability threat, especially for the Dia of 15 μm. Radial stress grows as the Dia decreases when the Dia is greater than 15 μm, and it decreases as the Dia decreases further (Figure 7b). Within a certain range of decreasing Dia, the distance reduces between the center of TSV filling Cu and the Si constraint surface, making it more difficult for radial stress inside the Cu to be released, resulting in the increment of radial stress. Dia reduction up to a certain threshold implies a decrease in the amount of Cu filling, and the influence of Cu on radial stress weakens. The axial stress varies positively with an Asp less than 4 and approximately stabilizes at an Asp exceeding 4 (Figure 7c). Within a certain range of increasing Asp, there is an increase in the confined area of Si, leading to the augmentation of axial stress. Once beyond this specific range of Asp, axial stress saturates consistently the von Mises stress trend. Except for the Dias of 10 and 15 μm, the axial stress is negatively correlated with the Dia. When the Dia is 10 μm, the maximum axial stress appears on the constraint surface at the bottom, which is not comparable, and the corresponding curve should be eliminated. A decrease in Dia within a limited range gives rise to an increase in the axial deformation gradient, more difficult axial stress release in the interior, and the augmentation of axial compressive stress. Cu plays a weaker role in the axial stresses due to the decrement of Cu filling; the axial stress decreases as the Dia decreases to a certain limit. In Figure 7d, the maximum shear stress occurs at a Dia of 15 μm.

## 3. Variation Analysis of Carrier Mobility

**Figure 8:**
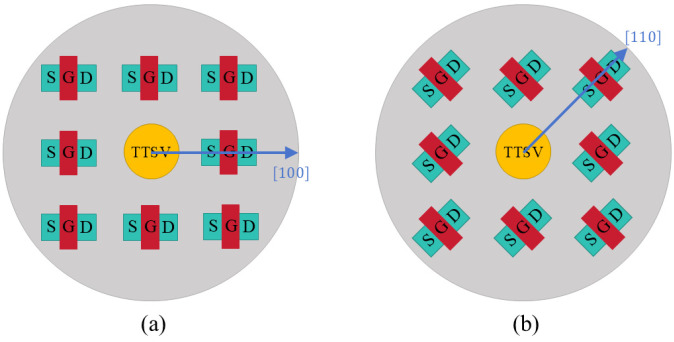
Schematic diagram of MOSFET distribution in the [100] (**a**) and [110] (**b**) channel directions around TTSV.

Most transistors are placed a few micrometers above the wafer surface. Since the deformation of the TTSV near the Si substrate surface is greater than in other areas, the thermal stress on the transistors near the TTSV deserves attention. Depending on the piezoresistive effect, due to the mechanical stress altering the resistivity of semiconductors, the resistivity of the Si substrate is subjected to TTSV thermal stress. The carrier mobility in Si versus TTSV thermal stress can be illustrated as (1) [37], where Δμ_ij_/μ, π_ijkl_, and σ_kl_ are the variation rate of carrier mobility, fourth-order tensor piezoresistive coefficient, and stress component, respectively. Due to the symmetry, stress-induced carrier mobility variation is simplified [38], where π_11_, π_12,_ and π_44_ correspond to the piezoresistive coefficient of the (100) crystal plane [39]. The subscripts of x, y, and z for both stress and carrier mobility represent the [100], [010], and [001] crystal orientations, respectively. According to (2), the variation in carrier mobility is not only influenced by the crystal plane of the Si substrate on which the active devices distribute but also by the orientation of the channel. Considering the technology process characteristics of MOSFET in 3D superconducting quantum chips, an analysis is conducted on the characteristics of carrier mobility variation for the Si (100) crystal plane, and the [100] and [110] crystal orientations (Figure 8). From (2), the transformation relationship between the variation rate of carrier mobility for the [100] and [110] crystal orientations and the thermal stress tensor are defined as (3) and (4), respectively.

(1) $$\left(\Delta \mu_{ij}/\mu \right)=-\pi_{ijkl}\sigma_{kl}$$

(2) $$\left[\begin{array}{c} \Delta \mu_{xx}/\mu_{xx}\\ \Delta \mu_{yy}/\mu_{yy}\\ \Delta \mu_{zz}/\mu_{zz}\\ \Delta \mu_{yz}/\mu_{yz}\\ \Delta \mu_{xz}/\mu_{xz}\\ \Delta \mu_{xy}/\mu_{xy} \end{array}\right]=\left[\begin{array}{cccccc} \pi_{11} & \pi_{12} & \pi_{12} & 0 & 0 & 0\\ \pi_{12} & \pi_{11} & \pi_{12} & 0 & 0 & 0\\ \pi_{12} & \pi_{12} & \pi_{11} & 0 & 0 & 0\\ 0 & 0 & 0 & \pi_{44} & 0 & 0\\ 0 & 0 & 0 & 0 & \pi_{44} & 0\\ 0 & 0 & 0 & 0 & 0 & \pi_{44} \end{array}\right]\left[\begin{array}{c} \sigma_{xx}\\ \sigma_{yy}\\ \sigma_{zz}\\ \sigma_{yz}\\ \sigma_{xz}\\ \sigma_{xy} \end{array}\right]$$

(3) $$(\Delta \mu /\mu {)}_{\left[100\right]}=-\pi_{11}\sigma_{xx}-\pi_{12}\sigma_{yy}-\pi_{12}\sigma_{zz}$$

(4) $$(\Delta \mu /\mu {)}_{\left[110\right]}=-\pi_{44}\sigma_{xy}$$

**Figure 9:**
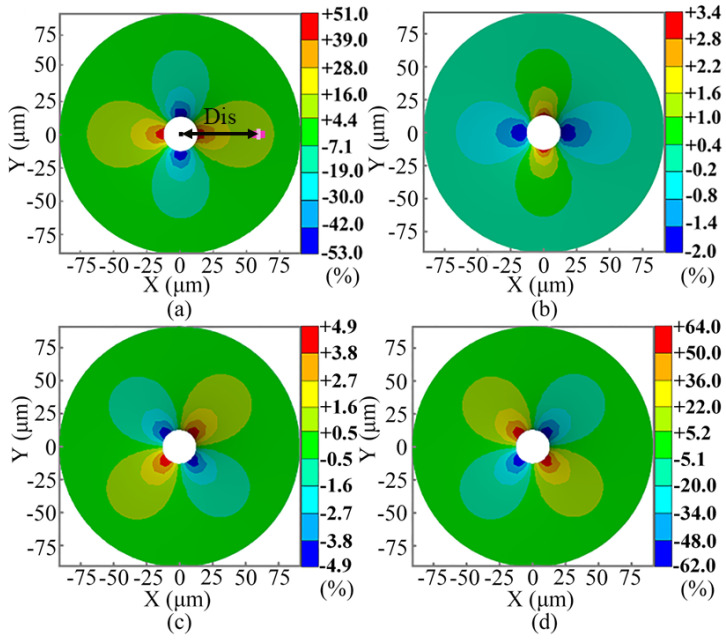
Contour plots of (**a**,**c**) electron and (**b**,**d**) hole mobility variations along the (**a**,**b**) [100] and (**c**,**d**) [110] crystal orientations.

The variation model of the near-surface carrier mobility of a single TTSV exhibits the same 3D characteristics as the thermal stress distribution model. To accurately assess carrier mobility variation, a TTSV model with a Dia of 25 μm and an Asp of 8 was selected for 3D FEA. By utilizing (3) and (4), the electron and hole mobility variation distributions in [100] and [110] crystal orientations induced by the thermal stress from the TTSV is obtained, as depicted in Figure 9, exhibiting double symmetry along both X/Y and XY axes, respectively. In the orthogonal direction, the magnitudes are similar, but the signs are opposite, with carrier mobility variations negatively correlated to distance. For the [100] crystal orientation, the maximum absolute value of electron mobility variation of 51.0% exceeds that of the hole. The hole mobility variation in the [110] crystal orientation surpasses that of the electron, with the maximum absolute variation of 64.0%. Both the electron mobility in the [100] crystal orientation and the hole mobility in the [110] crystal orientation exceed the Keep-Out-Zone (KOZ) criteria (regions with carrier mobility variation exceeding 5% or 10%). Such significant variations in carrier mobility can greatly impact the saturation current of MOSFET devices.

**Figure 10:**
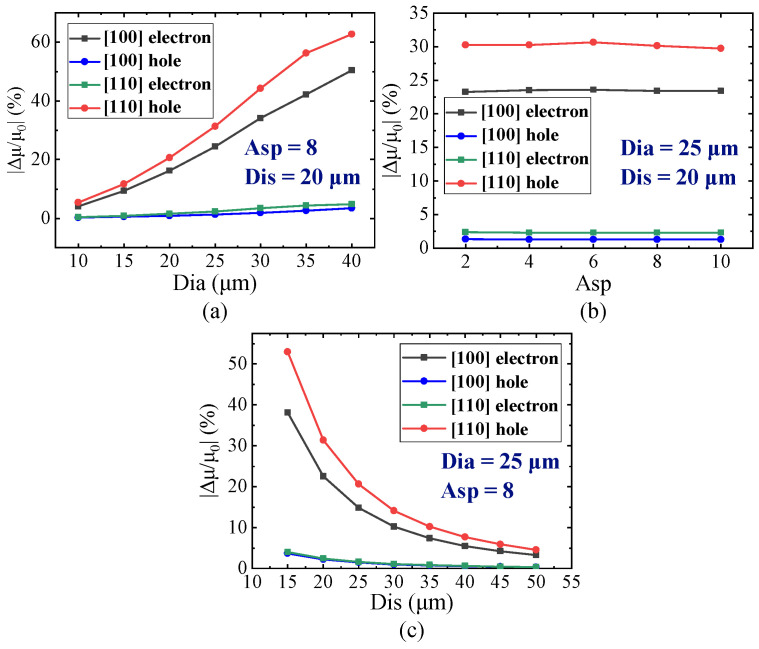
Influence of (**a**) Dia, (**b**) Asp, and (**c**) Dis on variation rate of carrier mobility.

**Figure 11:**
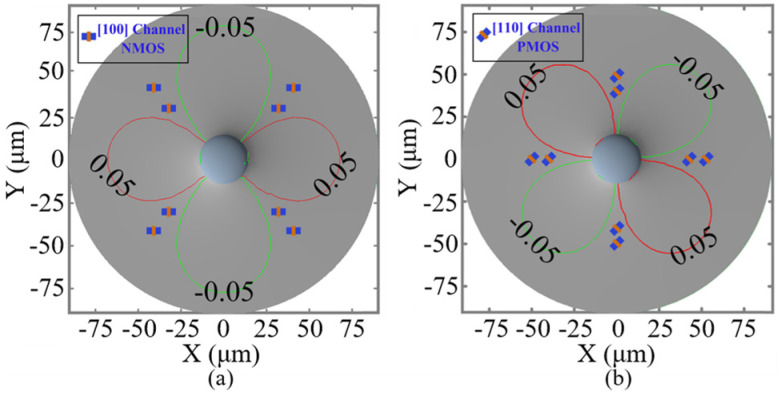
Optimal device placement of (**a**) NMOS in the [100] channel direction and (**b**) PMOS in the [110] channel direction.

Due to the near-surface positioning of MOSFET, it is imperative to analyze the impact of Dia and Asp, as well as the distance between the TTSV and MOSFET (Dis) (Figure 9a) on carrier mobility. As depicted in Figure 10a, while the Dis is 20 μm and the Asp is 8, the carrier mobility variations are positively correlated with the Dia, especially the electron mobility in the [100] crystal orientation and the hole mobility in the [110] crystal orientation, surging with the increase in Dia. There is no correlation between carrier mobility variation and Asp (Figure 10b). With a Dia of 25 μm and an Asp of 8, as illustrated in Figure 10c, the variation rate of carrier mobility changes inversely with Dis. Specifically, the electron mobility variation in the [100] crystal orientation and the hole mobility in the [110] crystal orientation experience significant reduction with an increase in Dis. The hole mobility in the [100] crystal orientation and the electron mobility in the [110] crystal orientation change little with Dia and Dis, and there are no corresponding KOZ around the TTSV. The P-Metal-Oxide-Semiconductor (PMOS) with a [100] channel direction and the N-Metal-Oxide-Semiconductor (NMOS) with a [110] channel direction can be arranged anywhere around the TTSV. The KOZs with the electron in the [100] crystal orientation and the hole in the [110] crystal orientation are larger in the X/Y and XY directions, respectively, and the mobility of the electron in the [100] crystal orientation and the hole in the [110] crystal orientation shifts greatly with Dia and Dis. For the optimal device arrangement, as shown in Figure 11, the NMOS with a [100] channel direction and the PMOS with a [110] channel direction near TTSV should be placed in XY and X/Y directions, respectively. For a small Dia or when the remote arrangement is allowed, it is advisable to orient NMOS and PMOS in a [110] and a [100] channel direction, respectively.

## 4. Electrical Performance Analysis of MOSFET

### 4.1. Saturation Current

**Figure 12:**
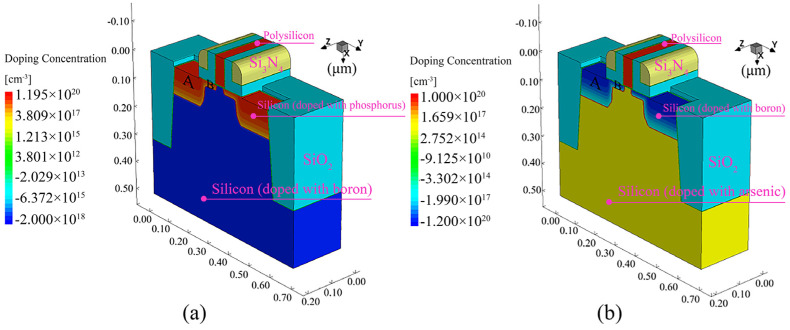
Structural profiles of (**a**) NMOS and (**b**) PMOS.

**Figure 13:**
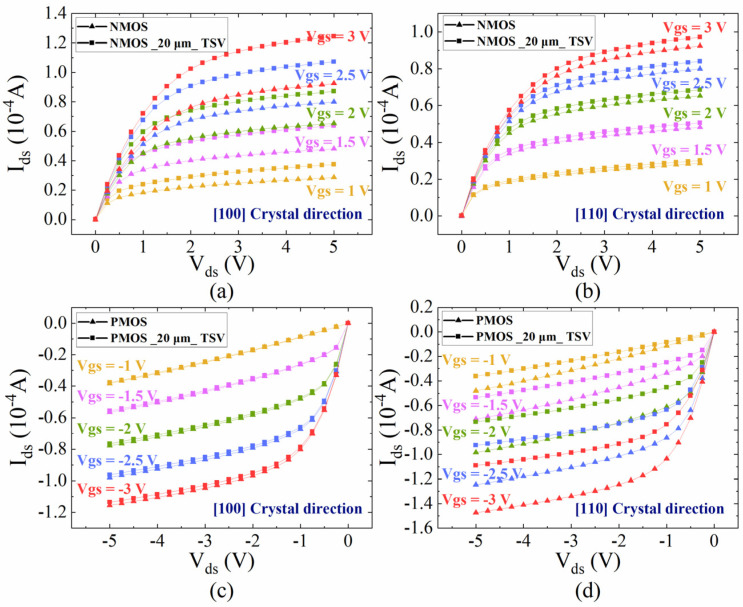
Characteristic curves with a Dia of 25 μm for (**a**,**b**) NMOS and (**c**,**d**) PMOS with (**a**,**c**) [100] and (**b**,**d**) [110] channel directions.

**Figure 14:**
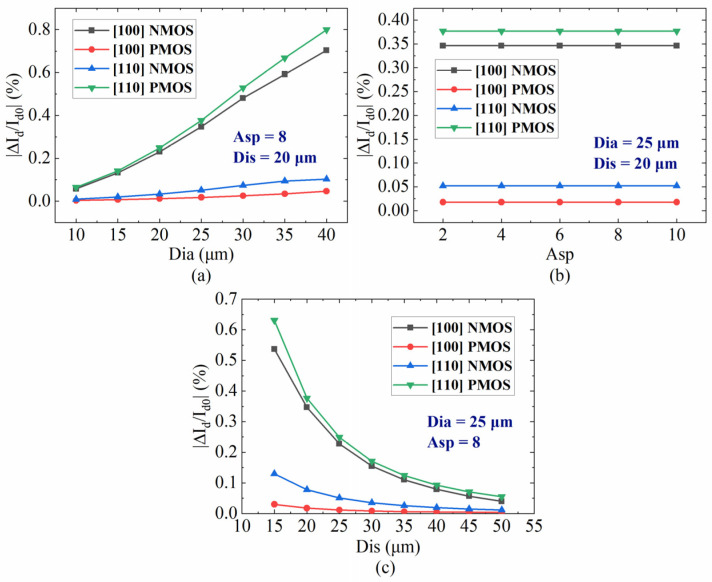
Effect of the (**a**) Dia, (**b**) Asp, and (**c**) Dis on the variation rate of the saturation current.

The nanoscale MOSFET models are established by device simulation tool Sentaurus to analyze the effect of carrier mobility variations provoked by TTSV thermal stress on the saturation current of MOSFET. The structural profiles of NMOS and PMOS are illustrated in Figure 12, and the characteristic parameters are presented in Table 1. For the 25 μm of Dia, Figure 13 plots the characteristic curves of the carrier mobility of NMOS and PMOS located 20 μm away from the TTSV at different gate voltages, where the deviation in drain current (I_ds_) depends on gate voltage (V_gs_), drain voltage (V_ds_), device type, and channel direction. For the NMOS with a [100] channel direction (Figure 13a) and the PMOS with a [110] channel direction (Figure 13d), the larger absolute values of V_gs_ or V_ds_ result in more pronounced curve deviations and greater changes in saturation current, reaching the maximum deviation of 34.3% at 3 V and 37.7% at −3 V for V_gs_. However, the saturation current remains relatively stable for the NMOS with a [110] channel direction (Figure 13b) and the PMOS with a [100] channel direction (Figure 13c). The deviation in saturation current resulting from TTSV thermal stress is directly linked to gate-level delay, and the cumulative effect of cell delays can significantly impact circuit-level delay [40]. Due to the serious drift of saturation current under the thermal stress of TTSV, further research is conducted on the effects of Dia, Asp, and Dis on the saturation current. As depicted in Figure 14a, the variation rate of the saturation current for the NMOS and PMOS with a Dis of 20 μm and Asp of 8 increases with an increase in the Dia, and the increase is more significant for the NMOS with the [100] channel direction and the PMOS with the [110] channel direction. The Asp demonstrates a minimal impact on the variation rate of the saturation current (Figure 14b). The rate decreases with an increase in Dis (Figure 14c), where the NMOS with the [100] channel direction and the PMOS with the [110] channel direction exhibit significant variations.

### 4.2. Threshold Voltage

**Figure 15:**
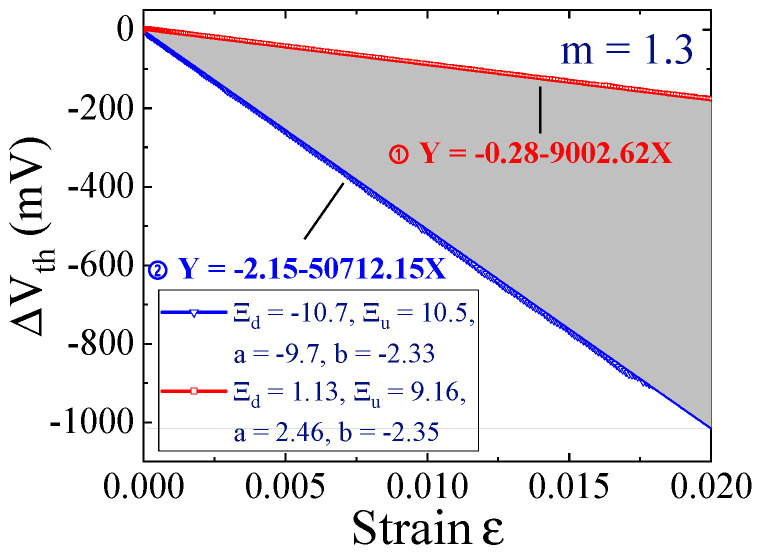
Functional relationship between the biaxial strain and threshold voltage.

According to the theory of deformation potential, the strain in semiconductors induces changes in energy levels, such as the splitting of bottom energy valley in the conduction band and the bending of the valence band and conduction band, causing changes in the surface potential of the MOSFET [41]. The threshold inversion point requires the surface potential of the device to reach twice the barrier height between the Fermi level and the local Fermi level. When there are changes in surface potential, it also affects the state of the threshold inversion point, leading to an alteration in the threshold voltage. The research conducted by Lim et al. [42] indicates that the biaxial strain causes a significantly larger drift in threshold voltage compared to the uniaxial strain. Figure 15 illustrates how the changing of threshold voltage depends on the biaxial strain [43], where m represents the effect coefficient of the volume, and Ξ_d_, Ξ_u_, a, and b denote the deforming potential coefficients. The function expression is fitted according to the two series of the deforming potential coefficients, and the filling between the two function curves represents the range of threshold voltage drift. Due to the wide range variation in the threshold voltage, as shown in Figure 15, the function of the upper and lower limit is used as the standard ① and ②, respectively.

**Figure 16:**
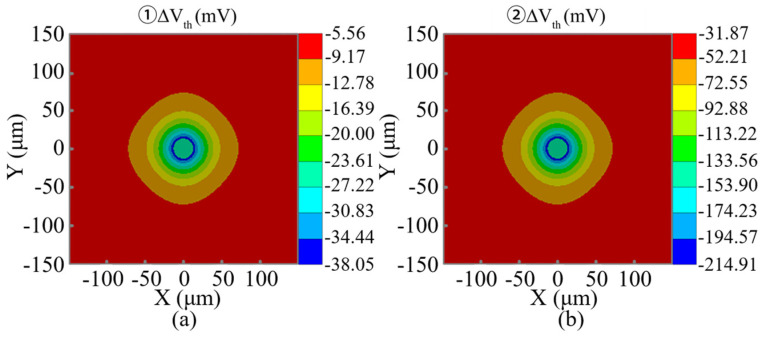
Threshold voltage variations under criteria (**a**) ① and (**b**) ②.

**Figure 17:**
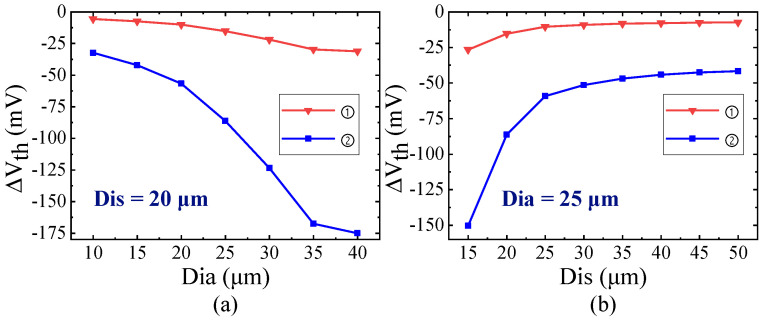
Impact of (**a**) Dia and (**b**) Dis on threshold voltage drift.

For the NMOS and PMOS devices affected by thermal stress from TTSV with a Dia of 25 μm, Figure 16 shows that the threshold voltage variation exhibits approximately double symmetry around the TTSV and the absolute values decrease as the Dis increases. At a Dis of 20 μm, the variation values of the threshold voltage for standard cases ① and ② are −9.96 and −56.65 mV, respectively. Due to the extensive coverage of TTSV stress compared to the single logic gate layout, the threshold voltage shifts in all transistors of the same type are uniform in magnitude [40,43]. Based on the superposition effect, even the minor change in threshold voltage induced by TTSV thermal stress can result in an exponential increase in gate leakage power and a wide range of circuit timing. Since this study focuses on a 2D analysis of the threshold voltage, the correlation with Asp is not investigated. The absolute value of threshold voltage variation in MOSFET is positively correlated with Dia for the Dis of 20 μm (Figure 17a) and negatively correlated with Dis for the Dia of 25 μm [see Figure 17b]. The effect trends of the Dia and Dis on carrier mobility, saturation current, and threshold voltage are the same.

## 5. Conclusions

Simulating the thermal condition within the refrigeration system and using temperature-dependent material properties, the thermal stress characteristics of TTSV in 3D-integrated chips with cooling down to ultra-low temperature are assessed. For the Asp of TTSV between 2 and 10, the dominant factor influencing thermal stress is the Dia. The TTSV deformation near the Si substrate surface is serious, and the carrier mobility of the Si substrate and the electrical characteristics of MOSFET around the TTSV vary significantly. In terms of the [100] and [110] crystal orientations, both electron and hole mobilities are pronouncedly affected by the variation in Dia and Dis, such as the change of 51.0% in electron mobility along the [100] crystal orientation. There is variation of approximately 34.3% in the saturation current at the 3 V of V_gs_ for the 45 nm-NMOS aligned with a [100] channel direction, and there is a change in threshold voltage as large as −56.65 mV in the 45 nm-MOSFET, which may affect power consumption and the logic function within the 3D chips. The carrier mobility, saturation current, and threshold voltage are all influenced by both Dia and Dis, following similar trends.

## Figures and Tables

**Table 1:** Characteristic parameters of NMOS and PMOS.

Type	Gate Length (nm)	Thickness of Gate Oxide Layer (nm)	Doping Concentration (cm^−3^)			Junction Depth (nm)	
			Channel	Zone A	Zone B	Zone A	Zone B
NMOS	45	15.37	−2.00 × 10^18^	7.34 × 10^19^	1.61 × 10^19^	87.95	18.14
PMOS	45	15.12	1.00 × 10^16^	−6.86 × 10^19^	−1.61 × 10^19^	77.20	15.78

## Data Availability

The data presented in this study are available upon request from the corresponding author.
